# Challenging Case: Robot-Assisted Laparoscopic Prostatectomy After Prior Suprapubic Open Prostatectomy

**DOI:** 10.1089/cren.2018.0015

**Published:** 2018-05-01

**Authors:** Shirin Razdan, Sanjay Razdan

**Affiliations:** ^1^ University of Miami Miller School of Medicine, Miami, Florida.; ^2^ International Robotic Prostatectomy Institute, Miami, Florida.

**Keywords:** prostate, prostate cancer, BPH, RALP, open prostatectomy

## Abstract

***Introduction:*** Given the ubiquity of robot-assisted laparoscopic prostatectomy (RALP) for treatment of localized prostate cancer, more surgeons are encountering challenging cases, either secondary to difficult anatomy, prior abdominal surgery, or prior radiation therapy. Our case is of RALP in a patient after prior suprapubic prostatectomy.

***Case Presentation:*** A 61-year-old otherwise healthy Hispanic gentleman presented for consultation after being found to have Gleason 4 + 4 = 8 prostate cancer on transrectal ultrasound-guided biopsy by an outside provider in July 2017. He had previously undergone suprapubic simple prostatectomy for benign prostatic hyperplasia (BPH) in Nicaragua more than a decade prior. The patient underwent RALP with bilateral nerve sparing in September 2017. The surgery was challenging in that extensive lysis of adhesions had to be performed and typical dissecting planes at the bladder neck and apex were distorted, insofar as meticulous care was taken to judiciously use thermal energy and rely on blunt dissection at these critical junctures. That being said, there were no operative or postoperative complications, the patient was discharged on postoperative day 1, and at 3-month follow-up, the patient was fully continent, maintained erections adequate for sexual intercourse, and had a prostate specific antigen <0.1. Pathology report returned Gleason 3 + 3 = 6 disease with negative surgical margins.

***Discussion:*** There is only one other example in the literature of RALP being performed after prior suprapubic prostatectomy. Our large RALP case volume (>5000 patients for a single surgeon and counting) provided us with the necessary experience required for encountering atypical anatomy, and thereby contributed to our patient's effective surgical outcome, both oncologic and functional.

***Conclusion:*** RALP for treatment of prostate cancer is a safe and appropriate option in men who have previously undergone suprapubic open prostatectomy for BPH, especially in the hands of an experienced surgeon.

## Introduction

Robot-assisted laparoscopic prostatectomy (RALP) has quickly become the preferred modality of treatment for localized prostate cancer. Given its ubiquity, more surgeons are encountering challenging cases, either secondary to difficult anatomy, prior abdominal surgery, or prior radiation therapy. One such scenario is RALP after surgical treatment of benign prostatic hyperplasia (BPH). Although medical treatment, such as with alpha blockers and five alpha reductase inhibitors, remains first-line therapy, in severe cases surgery becomes inevitable. Our patient underwent suprapubic open prostatectomy for BPH more than a decade ago in Nicaragua with removal of benign prostatic tissue. He was subsequently found in 2017 to have high-grade prostate cancer on transrectal ultrasound (TRUS)-guided biopsy and underwent RALP with bilateral nerve sparing in September 2017.

## Case Presentation

A 61-year-old otherwise healthy Hispanic gentleman presented for consultation after being found to have prostate cancer on TRUS-guided biopsy by an outside provider in July 2017. Biopsy revealed Gleason 4 + 4 = 8 adenocarcinoma of the prostate involving 50% to 90% of 3 of 12 cores, Gleason 4 + 3 = 7 adenocarcinoma involving 20% to 85% of 4 of 12 cores, and high-grade prostatic intraepithelial neoplasia. He was referred to an outside hospital for surgical removal of his prostate. However, given his history of suprapubic prostatectomy for BPH in Nicaragua more than a decade prior, he was advised to undergo radiation therapy. The patient approached us for second opinion and underwent pelvic MRI, bone scan, and cystoscopy for further characterization of his disease. MRI revealed diffuse heterogeneous postcontrast enhancement of the prostate gland with no discrete mass. There was no abnormal lymph node enhancement and bone scan was negative for metastatic disease as well. On cystoscopy, the patient was noted to have mild prostatic enlargement with a defect from his prior simple prostatectomy as well as a scar at the dome of the bladder, suggesting prior transvesical prostatectomy. Preoperative prostate specific antigen (PSA) was 9.2 and sexual health inventory for men (SHIM) score was 23.

Our patient underwent bilateral nerve sparing RALP in September 2017. Total operative time was 76 minutes. Blood loss was ∼25 mL. We used a transperitoneal four-arm approach with a 0° lens. Upon entry into the abdomen and colonic mobilization, care had to be taken while performing anterior bladder dissection because of adhesions present from the patient's prior open prostatectomy. After lysis of adhesions and dissection of the endopelvic fascia, the bladder neck was transected and the prostate was mobilized. A blunt posterior dissection was performed thereafter using PK scissors to minimize rectal damage. Dissecting planes at the bladder neck and the apex of the prostate were difficult and great caution had to be exercised to prevent positive margins or risk incontinence by not maintaining adequate urethral stump length. To that end, our modified maximal urethral length preservation (MULP) technique was employed to augment stump length. Bilateral neurovascular bundles were meticulously dissected away from the prostatic capsule using minimal thermal energy. Despite the inherently unfavorable anatomy, there were no operative complications and our patient was discharged on postoperative day 1 with an indwelling Foley catheter. He returned to clinic in 10 days for catheter removal. Pathology report returned back Gleason 3 + 3 = 6 adenocarcinoma of the prostate (downgraded) involving 25% of a 50 g prostate. Surgical margins and bilateral seminal vesicles were negative for cancer involvement. One pelvic lymph node was sampled and was negative for adenocarcinoma as well. Final staging of the disease was pT2pN0M0.

In December 2017, our patient returned for a 3-month follow-up appointment. At that time he endorsed perfect urinary continence with no pad or liner usage. In fact, he mentioned that he was fully continent as soon as 2 weeks after surgery. Our patient also stated that he was able to get and maintain erections strong enough for intercourse 1 month after surgery, with a postoperative SHIM score of 22. His PSA at the time of 3-month follow-up was <0.1 ng/mL.

## Discussion

Suprapubic simple prostatectomy is still a viable option for men with BPH and large adenomas (prostate size >100 cm^3^), especially after failure of medical management. The procedure is efficacious in providing long-term improvement in International Prostate Symptom Score, postvoid urine residual volume, and maximal flow rate. The incidence of simple prostatectomy has decreased in recent years, with the majority being performed as open procedures rather than minimally invasive.^1^ Although the overall incidence has decreased, >2000 cases are still performed annually using an open approach, about 5% of all prostatectomies performed in the United States, thereby making subsequent robotic prostatectomy for prostate cancer a real possibility.^2^

The literature includes copious examples of radical prostatectomy performed after surgical treatment for BPH, with the most common prior surgery being transurethral resection of the prostate. Clinical and functional outcomes after prior prostate surgery are significantly different than those in patients who are naive to surgery, with increased risk of blood loss, increased need for bladder neck reconstruction, and longer operative times. However, results are generally favorable in the hands of experienced surgeons, whether they employ a robotic technique or not, with equivocal differences in positive surgical margin status and biochemical recurrence rates.^3^

There is only one other reported case of RALP after prior suprapubic prostatectomy in the literature. Tsui et al. reported their case in 2016 with favorable results.^4^ At 9 weeks postoperative follow-up, their patient was continent and with a PSA <0.01 ng/mL; it was too soon to assess erectile function. Tsui et al.'s approach to the RALP was different than our approach in that they relied on ureteral stent placement after bladder neck incision to mark the positions of the ureteral orifices because of altered anatomy. We chose not to employ this technique, as the orifices were easily visible. Otherwise, in our approach to RALP after open prostatectomy, we experienced similar challenges of hostile anatomy and took comparable precautions in ensuring we avoided positive surgical margins and maintained adequate oncologic and functional outcomes. To this end, our large RALP case volume (>5000 patients for a single surgeon and counting) provided us with the necessary experience required for encountering atypical anatomy, and thereby contributed to our patient's effective surgical outcome. We recommend that when treating a patient with prostate cancer who has had prior open prostatectomy for BPH, surgeon experience should be taken into account to ensure outcomes comparable with those of patients who have had no prior prostate surgery.

## Conclusion

RALP for treatment of prostate cancer is a safe and appropriate option in men who have previously undergone suprapubic open prostatectomy for BPH, especially in the hands of an experienced surgeon.

## Abbreviations Used

BPH: benign prostatic hyperplasia
MRI: magnetic resonance imaging
PSA: prostate specific antigen
RALP: robot-assisted laparoscopic prostatectomy
SHIM: sexual health inventory for men
TRUS: transrectal ultrasound
